# Can mutations in *ELA2*, neutrophil elastase expression or differential cell toxicity explain sulphasalazine-induced agranulocytosis?

**DOI:** 10.1186/1471-2326-4-5

**Published:** 2004-12-02

**Authors:** Annica Jacobson, Håkan Melhus, Mia Wadelius

**Affiliations:** 1 Department of Medical Sciences, Uppsala University, Uppsala University Hospital S- 751 85 Uppsala, Sweden

## Abstract

**Background:**

Drug-induced agranulocytosis, a severe side effect marked by a deficit or absolute lack of granulocytic white blood cells, is a rare side-effect of the anti-inflammatory drug sulphasalazine. Mutations in the human neutrophil elastase gene (*ELA2*), causing increased intracellular concentration of this serine protease, inhibits neutrophil differentiation in severe congenital neutropenia (SCN). Since the clinical symptoms of agranulocytosis and SCN are similar, we hypothesized that it may origin from a common genetic variation in *ELA2 *or that sulphasalazine may affect human neutrophil elastase activity and protein expression.

**Methods:**

We screened for genetic differences in *ELA2 *in DNA from 36 patients who had suffered from sulphasalazine-induced agranulocytosis, and compared them with 72 patients treated with sulphasalazine without blood reactions. We also performed in vitro studies of the blood cell lines HL60 and U937 after sulphasalazine exposure with respect to cell survival index, neutrophil elastase protein expression and activity.

**Results:**

None of the mutations in *ELA2*, which previously have been reported to be associated with SCN, was found in this material. Protein expression of human neutrophil elastase in lymphoma U937 cells was not affected by treatment with concentrations equivalent to therapeutic doses. Cell survival of lymphoma U937 and promyelocytic leukemia HL-60 cells was not affected in this concentration range, but exhibited a decreased proliferative capacity with higher sulphasalazine concentrations. Interestingly the promyelocytic cells were more sensitive to sulphasalazine than the lymphoma cell line.

**Conclusion:**

Neutrophil elastase expression and *ELA2 *mutations do, however, not seem to be involved in the etilogy of sulphasalazine-induced agranulocytosis. Why sulphasalazine is more toxic to promyelocytes than to lymphocytes remains to be explained.

## Background

Sulphasalazine (SA) has anti-inflammatory, immunosuppressive and antibiotic actions, and is a component in the therapy of Crohn's disease, ulcerative colitis and rheumatoid arthritis. Bacterial enzymes in the colon split sulphasalazine into sulphapyridine and 5-aminosalicylic acid before it is absorbed. Sulphapyridine acts as a sulphonamide antibiotic, whereas 5-aminosalicylic acid is believed to be the anti-inflammatory metabolite. Common side/toxic effects are vomiting, skin rash and headache. The incidence of the hematological adverse effects associated with sulphasalazine is generally low, but the reactions can be severe and sometimes fatal. The risk of sulphasalazine-induced agranulocytosis, i.e. profoundly depressed circulating neutrophils is highest within the first three months of sulphasalazine-treatment, with a fatality rate of 6.5 % [1]. Clinical symptoms of agranulocytosis include fever, malaise and susceptibility to infections. Patients with arthritic disorders have a greater risk of developing sulphasalazine-induced agranulocytosis than patients with inflammatory bowel diseases.

Severe congenital neutropenia (SCN) and cyclic neutropenia (CN) occur both as inherited and as sporadic diseases. SCN has a constant low neutrophil number if left untreated, whereas CN manifests with cyclic oscillations of neutrophil number with a 21-day cycle. Recently, diverse heterozygous mutations in *ELA2*, encoding human neutrophil elastase, have been identified in a majority of the cases with CN and two-thirds of the cases with SCN [2].

In this study, we hypothesized that sulphasalazine-induced agranulocytosis, with clinical symptoms similar to congenital neutropenia, may arise from genetic variation in the human neutrophil elastase gene. We genotyped 108 sulphasalazine-treated patients for *ELA2*, one third which of had experienced sulphasalazine-induced agranulocytosis. We, furthermore, tested for cytotoxic doses of sulphasalazine, and studied protein expression of human neutrophil elastase in sulphasalazine-treated blood cell lines.

## Methods

### Subjects

Patients were treated with sulphasalazine (Salazopyrin, Pharmacia, Sweden) for inflammatory joint diseases and inflammatory bowel disease. The cases with sulphasalazine-induced agranulocytosis were originally collected through the Swedish Medical Products Agency's register of adverse side effects [3]. The control group had been treated with sulphasalazine without adverse effects for at least 3 months. From the original patient material consisting of 39 cases and 75 controls, DNA was available for 36 cases and 72 controls. The patient journals were studied for information concerning neutrophil differentiation in bone marrow aspirates. The study was approved by the Ethics Committee of the Medical Faculty at Uppsala University, registration number 95–200.

### Mutation analysis

**Figure 1:**
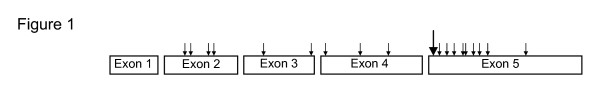
**Outline of reported mutations in ELA2 exon-sequences in patients with severe congenital neutropenia and cyclic neutropenia **Outline of mutations previously reported [2, 4] in *ELA2 *exons 1–5. The SNP S173 [6] is indicated as an extended arrow and represents base number 4890 in accession number Y00477 and is a base C→A substitution.

Genomic DNA was extracted from whole blood using standard techniques. Fragments covering exons 2–5 of *ELA2 *were amplified by PCR using primer pairs listed in Table 1. The selection of exons 2–5 and some of the flanking intron sequences was based on previously reported mutations in cases with SCN and CN [2,4], as outlined in Figure 1. Products for exon 2–5 were amplified with 1.5 units of AmpliTaq Gold DNA polymerase (Applied Biosystems), activated by 15 min at 95°C followed by 4 cycles 94°C 30 sec, 65°C 30 sec, 72°C 1.5 min and 35 cycles of 94°C 30 sec, 67°C 30 sec, 72°C 1.5 min with a final extension of 10 min at 72°C. The exception was amplification of exon 5, where a 2°C lower annealing temperature was used. All primers contained a consensus M13 sequence to enable sequencing with the same primer, included in BigDye Primer sequencing kit from Applied Biosystems, Stockholm, Sweden. Applied Biosystems 310 analyzer and Sequence Analysis software was used for all sequencing. Thus, the 36 cases and 72 controls were analyzed for genetic mutations in *ELA2*.

**Table 1:** Primer sequences for PCR amplification of *ELA2 *exon 2–5

*ELA2 *target sequence	Primers
**Exon 2 F**	5'-tgtaaaacgacggccagtgggaggggacaggctccttgg-3'
**Exon 2 R**	5'-caggaaacagctatgaccaccgggacgcggggtccgagc-3'
**Exon 3 F**	5'-tgtaaaacgacggccagtcaggcccgtcgccggatggg-3'
**Exon 3 R**	5'-caggaaacagctatgacctccgtcgcagcctccaccct-3'
**Exon 4 F**	5'-tgtaaaacgacggccagtgtgacgcgctgacgatctgt-3'
**Exon 4 R**	5'-caggaaacagctatgaccgcagtaccgggctgggagcg-3'
**Exon 5 F**	5'-tgtaaaacgacggccagtcagtccagcttccccacctt-3',
**Exon 5 R**	5'-caggaaacagctatgaccgacctactgaccattttcaac-3'

PCR primers sequences for *ELA2 *exon 2–5, for following sequencing reactions with BigDye primer, Applied Biosystems.

### Cell culture

The lymphoma cell line U937 and the promyelocytic cell line HL-60 (American tissue culture collection) were cultured in Dulbeccos modified Eagles Medium, DMEM (Sigma) supplemented with 10 % fetal bovine serum (SVA, Uppsala, Sweden), L-glutamine and penicillin-streptomycin (Sigma).

### Western blot

Equal numbers (8 × 10^6^) of U937 cells were grown in 75 cm^2 ^dishes in complete medium containing 0, 125 and 250 μM sulphasalazine for 24 h. For protein isolation, cells were washed in PBS and lysed in buffer containing 1% Triton X-100, 50 mM Tris-HCl pH 8.0 and protease inhibitor cocktail (Sigma) and were kept on ice for 30 min. Lysates were centrifuged for 10 min at 10 000 × g, and protein concentration was determined using BioRad protein assay. Criterion precast gels (BioRad, Sweden) were used to perform SDS-page with 20 μg protein loaded per well. After gel transfer to a nitrocellulose membrane, the membranes were blocked over night in 5 % dry milk in TBS-Tween. Primary antibody against human neutrophil elastase (Calbiochem, Sweden) was diluted 1:1000 in 5 % dry milk in TBS-T. After 2 h incubation, and four sets of washing, a secondary antibody was added (1:5000) and blots were developed using ECL (ECL Western blotting system, Amersham, Sweden). Western blot analysis of human neutrophil elastase expression was performed twice.

### Elastase activity assay

Cells (HL-60 and U937) treated with 0, 125, 250 and 500 μM of for 24 h were lysed with 100 μl of buffer containing 100 mM Tris-HCl pH 7.4, 1 mM MgCl_2_, 0.1 % Triton X-100. After homogenization, 300 μl of 1.4 M NaCl in 0.1 % Triton X-100 was added and samples were centrifuged at 15 000 × g, for 15 min at 4°C. The supernatants were transferred to new tubes and assayed for elastase activity using Suc-Ala-Ala-Ala-pNA (Sigma) as a substrate. For each assay we took 25 μl sample, mixed with 100 μl buffer containing 100 mM Tris-HCl pH 8.5, 1 M NaCl, 500 mM MgCl_2 _and 0.1 % Triton X-100. To this, 50 μl of substrate was added, to a final concentration 1 μM. After 30 min of incubation in room temperature, absorbance was read at 405 nm and the concentration was calculated from a standard curve of elastase (Sigma).

### Cell survival index

For the cell viability assay, we used a fluorometric microculture cytotoxicity assay (FMCA) previously described by Larsson et al [5]. Briefly, 20 000 cells/well were plated in 96-well plates (NUNC, DK) in complete medium with addition of increased concentrations of sulphasalazine (0, 125, 250, 500, 750 and 1000 μM) and incubated for 72 h in a humidified atmosphere used in regular cell culturing. All samples were plated in triplicates and three wells with cell culture medium served as blanks. As controls we had cells without additions and cells only with solvent, in this case 0.5 M NaOH, with equal molarities as in the wells with the highest sulphasalazine-concentration. At the end of the 72 h incubation period, plates were centrifuged (200 × g, 5 min) and medium was aspirated in a microtitre plate washer, washed with PBS and 100 μl of 10 μg/ml of fluorescein diacetate (Sigma, Sweden), was added. This dye exclusively binds intact cell membranes of viable cells. After 1 h incubation at 37°C, the fluorescence was read in the Fluoroscan 2 (Labsystems OY, Finland) at 480 nm excitation and 530 nm as emission. The results are presented as survival index, defined as fluorescence in test wells/ fluorescence in control wells (blank values subtracted) × 100. Thus, a low numerical value indicates high sensitivity to the cytotoxic effect of sulphasalazine. Effective concentration is defined as the concentration when 50 % of the cells are viable (EC_50_).

### Statistics

Two-tailed Student's t-test was used to compare subject characteristics and results from cell culture between cases and controls. Frequencies of subject characteristics male versus females was tested with Chi^2^-test with one degree of freedom, using Minitab 14. A *p*-value less than 0.05 was denoted with (*), *p *< 0.01 with (**) and was considered as statistically significant.

## Results

### Subjects

The characteristics of the subjects are presented in Table 2. The agranulocytosis cases were significantly older than the control patients (*p *= 0.023). The white blood cell count (WBC) before sulphasalazine-treatment did not differ between cases and controls, nor did the dose of sulphasalazine. Bone marrow aspirates had been taken from 10 patients (cases). In all samples, the myelopoesis was seriously reduced and a maturation arrest at the promyelocyte-myelocyte stage of neutrophilic differentiation was seen.

**Table 2:** Characteristics of subjects

	Cases (n = 36)	Controls (n = 72)	*p*-value
**Age range (median)**	11–77 (55)	13–90 (47)	0.023
**WBC before ^a^**	9.3 ± 4.7	8.5 ± 2.5	0.26
**Dose of sulphasalazine (gram/day)**	2.2 ± 0.6	2.0 ± 0.4	0.13
**Male : Female**	17 : 19	33 : 39	0.891

^a ^WBC data only available for 28 cases and 67 controls.

Cases are defined as the patients treated with sulphasalazine, who were diagnosed with agranulocytosis and controls were defined as patients treated with sulphasalazine without hematological side effects within the first three months of sulphasalazine treatment. The statistics of differences between cases and controls in age, white blood cell count (WBC) before sulphasalazine treatment and dose of sulphasalazine was calculated with 2-tailed Student's t-test. The frequency of males versus females was calculated with Chi^2 ^test, one degree of freedom. Significance level was set at 0.05.

### Mutation analysis

None of the previously reported mutations in *ELA2 *was found in this material, although we found a silent single nucleotide polymorphism, called S173 [6] that corresponds to a C4890A substitution in Genbank accession number Y00477 (marked with extended arrow in Figure 1). The incidence of the S173 polymorphism did not differ between controls and cases, 0.31 for both, and S173 has previously been detected in healthy subjects [6]. No correlation between the S173 polymorphism and white blood count before sulphasalazine-treatment was found (Table 3).

**Table 3:** White blood count (WBC), before sulphasalazine treatment, in subjects with or without the S173 polymorphism

	**WBC**
Subjects with S173 (n = 34)	8.74 ± 2.51
Subjects without S173 (n = 74)	8.66 ± 3.51

White blood count (WBC) was estimated by the local physicians before starting with the sulphasalazine treatment. The average WBC in subjects with or without S173 is presented as the mean value ± SD. It was no statistical difference between the groups.

### Elastase expression, elastase activity and cell survival after sulphasalazine exposure to HL-60 and U937

**Figure 2:**
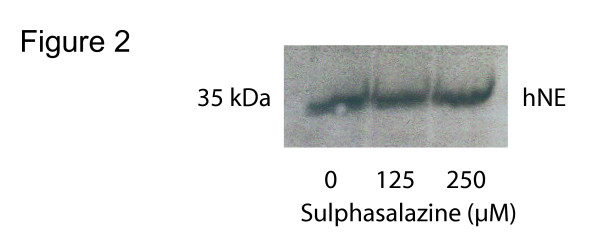
**Western blot analysis of human neutrophil elastase (hNE) expression after sulphasalazine exposure **U937 cells were incubated for 24 h with 0, 125 and 250 μM sulphasalazine, followed by cell lysis and protein isolation. 20 μg of protein was applied in each lane, transferred to nitrocellulose membrane and incubated with human neutrophil elastase antibody.

**Figure 3:**
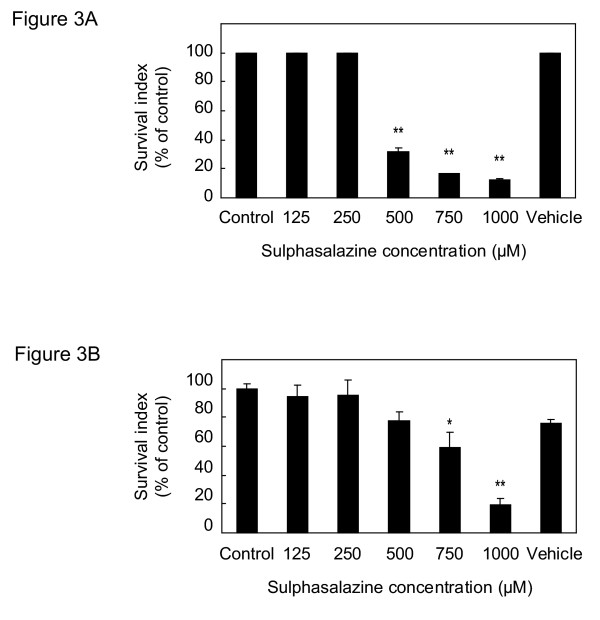
**Survival index of HL-60 and U937 cells, incubated with increasing concentrations of sulphasalazine **Survival index of HL-60 (A) and U937 cells (B) treated with increasing concentrations of sulphasalazine (0–1000 μM) for 72 h and measured with FMCA. Survival index, defined as fluorescence in test wells/ fluorescence in control wells (blank values subtracted) × 100.

By western blot analysis, we analyzed neutrophil elastase protein expression in U937 cells. No difference in human neutrophil elastase expression was detected after treatment with 125 and 250 μM sulphasalazine (Figure 2), compared to controls. The elastase activity in HL-60 and U937 cells was not affected by increasing sulphasalazine concentration, ranging from 0 to 500 μM, and expressed as elastase activity/μg protein (data not shown). For the cell survival index, each FMCA experiment was performed three times separately with similar results (inter-assay variation less than 10 %). Concentrations below 250 μM sulphasalazine did not affect the survival index of U937 and HL-60 cells (Figure 3A,3B), but at 500 μM of sulphasalazine, the survival index of HL-60 cells decreased to a third (Figure 3A). The U937 was only marginally affected at 500 μM sulphasalazine-concentration, but cellular survival decreased with approximately 40 % at 750 μM of sulphasalazine (Figure 3B). The effective concentration (EC_50_) of sulphasalazine was approximately 370 μM for HL-60 cells and 820 μM for U937 cells.

## Discussion

Idiosyncratic drug-induced agranulocytosis can be due to several different mechanisms of action, including immunological, toxic and genetic [7,8]. Toxic drug-induced neutropenia is often dose-dependent, whereas immunological and genetic causes are less related to dose. In our study, bone marrow aspirates from patients with sulphasalazine-induced agranulocytosis revealed maturation arrest of neutrophils at the promyelocyte-myelocyte stage. These findings resemble promyelocytic maturation arrest seen in severe congenital neutopenia (SCN) and cyclic neutropenia (CN) [9]. In the majority of cases with SCN and CN, germline mutations in the human neutrophil elastase gene (*ELA2*) are implicated as the primary abnormality [2,4]. The focus of this study is therefore on the human neutrophil elastase gene as a possible cause of sulphasalazine-induced agranulocytosis. We found a coding synonymous polymorphism in *ELA2*, which, however, was equally represented among cases and controls.

Heterozygous mutations in *ELA2 *act in a dominant manner, interfering with sub-cellular trafficking of neutrophil elastase, and leading to an accumulation of neutrophil elastase in the cytosol [10]. For normal neutrophil cell maturation, the proliferative action of the granulocyte colony stimulating factor (G-CSF) is necessary [11]. When G-CSF is exposed to active elastase enzyme in vitro, G-CSF is rapidly cleaved and rendered inactive [11]. In theory, SCN and CN are caused by an accumulation of neutrophil elastase, leading to an inactivation of G-CSF and a negative feedback on granulopoiesis, which causes neutropenia. Other proteins, connected to expression and transportation of human neutrophil elastase, have also been linked to SCN disease. In canine cyclic hematopoieses, lack of the intracellular transport protein AP3β causes accumulation of canine neutrophil elastase in the cytosolic compartments [12], and mutations in *ELA2 *may disrupt the AP3β-recognition site [13]. Furthermore, mutations in the proto-oncogene *GFI1*, a transcriptional repressor of *ELA2*, causes over-expression of neutrophil elastase in mice, thus, making them neutropenic [14].

During maintenance therapy with sulphasalazine, trough serum sulpha concentration is on average approximately 100 μM at the Department of Clinical chemistry and pharmacology, Uppsala University hospital. To avoid toxic effects, trough serum concentration of sulpha should stay below 600 μM [15]. Our in vitro data suggest a decreased cell survival of sulphasalazine at concentrations around 500 μM. Interestingly promyelocytic leukemia HL-60 cells were more sensitive to sulphasalazine than lymphoma U937 cells, with EC_50 _values of 370 μM and 820 μM, respectively. Human neutrophil elastase expression in lymphoma U937 cells did not differ after sulphasalazine at 125 and 250 μM, indicating that human neutrophil elastase production is not affected by sulphasalazine at subtoxic levels.

## Conclusions

In conclusion, neutrophil elastase does not appear to be involved in the etiology of sulphasalazine-induced agranulocytosis. No causative *ELA2 *mutations were found, and therapeutic concentrations of sulphasalazine did not increase the expression of human neutrophil elastase. High concentrations of sulphasalazine were toxic to white blood cells in vitro; however, there is no evidence that this toxicity is mediated through human neutrophil elastase. Promyelocytic cells were more sensitive to sulphasalazine than lymphoma cells, and the reason for this difference may also explain sulphasalazine-induced agranulocytosis.

## Competing interests

The author(s) declare that they have no competing interests.

## Authors' contributions

AJ carried out the molecular genetic studies, participated in the sequence alignment, drafted the manuscript and carried out the in vitro experiments. MW participated in the design of the study and performed the statistical analysis. HM conceived the study, and AJ, MW and HM participated in its design and coordination. All authors read and approved the final manuscript.

## Pre-publication history

The pre-publication history for this paper can be accessed here:



## References

[B1] Keisu M, Ekman E, Wiholm BE (1992). Comparing risk estimates of sulphonamide-induced agranulocytosis from the Swedish Drug Monitoring System and a case-control study. Eur J Clin Pharmacol.

[B2] Ancliff PJ, Gale RE, Liesner R, Hann IM, Linch DC (2001). Mutations in the ELA2 gene encoding neutrophil elastase are present in most patients with sporadic severe congenital neutropenia but only in some patients with the familial form of the disease. Blood.

[B3] Wadelius M, Stjernberg E, Wiholm BE, Rane A (2000). Polymorphisms of NAT2 in relation to sulphasalazine-induced agranulocytosis. Pharmacogenetics.

[B4] Dale DC, Person RE, Bolyard AA, Aprikyan AG, Bos C, Bonilla MA, Boxer LA, Kannourakis G, Zeidler C, Welte K, Benson KF, Horwitz M (2000). Mutations in the gene encoding neutrophil elastase in congenital and cyclic neutropenia. Blood.

[B5] Larsson R, Kristensen J, Sandberg C, Nygren P (1992). Laboratory determination of chemotherapeutic drug resistance in tumor cells from patients with leukemia, using a fluorometric microculture cytotoxicity assay (FMCA). Int J Cancer.

[B6] Horwitz M, Benson KF, Person RE, Aprikyan AG, Dale DC (1999). Mutations in ELA2, encoding neutrophil elastase, define a 21-day biological clock in cyclic haematopoiesis. Nat Genet.

[B7] Palmblad J, Papadaki HA, Eliopoulos G (2001). Acute and chronic neutropenias. What is new?. J Intern Med.

[B8] van Staa TP, Boulton F, Cooper C, Hagenbeek A, Inskip H, Leufkens HG (2003). Neutropenia and agranulocytosis in England and Wales: incidence and risk factors. Am J Hematol.

[B9] Zeidler C, Welte K (2002). Kostmann syndrome and severe congenital neutropenia. Semin Hematol.

[B10] Li FQ, Horwitz M (2001). Characterization of mutant neutrophil elastase in severe congenital neutropenia. J Biol Chem.

[B11] El Ouriaghli F, Fujiwara H, Melenhorst JJ, Sconocchia G, Hensel N, Barrett AJ (2003). Neutrophil elastase enzymatically antagonizes the in vitro action of G-CSF: implications for the regulation of granulopoiesis. Blood.

[B12] Benson KF, Li FQ, Person RE, Albani D, Duan Z, Wechsler J, Meade-White K, Williams K, Acland GM, Niemeyer G, Lothrop CD, Horwitz M (2003). Mutations associated with neutropenia in dogs and humans disrupt intracellular transport of neutrophil elastase. Nat Genet.

[B13] Horwitz M, Benson KF, Duan Z, Li FQ, Person RE (2004). Hereditary neutropenia: dogs explain human neutrophil elastase mutations. Trends Mol Med.

[B14] Person RE, Li FQ, Duan Z, Benson KF, Wechsler J, Papadaki HA, Eliopoulos G, Kaufman C, Bertolone SJ, Nakamoto B, Papayannopoulou T, Grimes HL, Horwitz M (2003). Mutations in proto-oncogene GFI1 cause human neutropenia and target ELA2. Nat Genet.

[B15] Rieder J, Schwartz DE, Fernex M, Bergan T, Brodwall EK, Blumberg A, Cottier P, Scheitlin W (1974). Pharmacokinetics of the antibacterial combination sulfamethoxazole plus trimethoprim in patients with normal or impaired kidney function. Antibiot Chemother.

